# Dentistry: Past, Present and Future

**Published:** 1893-10

**Authors:** A. C. Cogswell

**Affiliations:** Halifax, N. S.


AMERICAN JOURNAL

—OF—

DENTAL SCIENCE.

Vol. XXVII. Baltimore, October, 1893. No. 6.

ARTICLE I.

### DENTISTRY: PAST, PRESENT AND FUTURE.

BY A. C. COGSWELL, D. D. S., HALIFAX, N. S.

*Mr. President and Gentlemen:*

I have chosen for my paper, to be read before your second annual Dental Association meeting, this subject, "Dentistry: Past, Present and Future."

Possibly you will indulge me by allowing one who has devoted the past forty years of his life in introducing in this paper some personal reminiscences and experiences during the past two decades in connection with dentistry. While I desire to refer to the profession in the past, present and future, I may be allowed to review from journals, periodicals and memory, some things that may be of interest to many in the profession. I must remind you, however, that those starting now in the practice of dentistry have far greater advantages and opportunities than those of my day, forty years ago, and, from this standpoint, we may look to the future for greater advancement than in the past, and "from those who have much, much will be required."

Of the remote past, history has given us little light on the methods adopted many centuries ago to alleviate pain and suffering caused from diseased teeth. Some 460 years B. C., Hippocrates refers to fixing the teeth, probably some dental operation. In the sixth century, Celsus and others had vague ideas of the treatment of the dental organs. In 1747, dentistry was practised by John Hunter, and in 1786 by Bourdett. In 1800 it was practiced more commonly. During a visit to Washington, D. C., in the year 1865, I examined a set of teeth made for and worn by George Washington, the first President of the United States of America. The case represented a complete set of human teeth on bone plate, to which were attached gold spiral syrings. The work had been done somewhere between the years 1782 and 1792, as Washington was born in 1732, inaugurated President in 1790, and died at the age of sixty-seven in the year 1799, and we may conclude that the work was not less than at least 100 years old, or made about the year 1792.

In 1800, barbers, watchmakers and cheap mechanics practiced upon teeth for simply the money to be obtained for a livelihood, and decoyed many, like the spider and the fly, by advertisements and circulars, by inviting them to walk into their parlors, where all kinds of work was done at the cheapest possible rate. In 1851, while on a visit to St. Louis, my attention was directed to a sign on one of the principal streets, which read as follows: "Cupping, Leaching, Tooth-pulling and Barbering done here;" and in front of the sign was the barber's pole. Coming down still later, in the year 1890, a gentleman passing along one of the principal thoroughfares in the great city of New York, had two handbills presented to him, which set forth the qualifications and abilities of the first as a professional boot-black; the second, the dentist, as a business man.

The first circular read as follows: "Professor Johnson, specialist, begs to call your attention to his parlors on 49 Cortland Street, N. Y., where you may have the encasements of your *pedal* extremities illuminated or lubricated for the Infinitesimal sum of five cents, in a truly scientific manner. The place is lighted throughout by incandescent light; the boys are polite, and work with elastic rapidity. Your valise, bag, cane or umbrella checked at all times; try me." The second circular, headed "Dentistry," was as follows: "Dr. W. F. Nistop, Dentist, No. 999 15th Avenue, N. Y., for nearly twenty years with Dr. Dear, of this city, begs to announce that he has established himself at the above address, with every facility for doing a first-class dental business in all its various branches, at the following low rates: Teeth extracted, 25 cents; Gas, 50 cents; Gold fillings, $1 upwards (with gas 50) according to labor. Best artificial sets at cheapest rates, only the best material used, satisfaction guaranteed. Fresh laughing gas daily. Open from 7 a. m. to 8 p. m. . Sundays all day. Call and get my prices before going elsewhere." One would almost imagine that some of the advertisements seen in our Dominion papers within the past few years or at the present time, had been carefully copied from the above, especially the latter part. A writer signing himself D. D. S., in the *Dental Advertiser* of 1890, referring to the above advertisements or circulars, remarks as follows: "Not any wonder that the dentist dies poor, and that the patients regard him as a very common sort of a person, when the dentist himself calls his profession a business, and the boot-black his business a profession. Which of the two is going up in the scale? and which of the two is likely to have a monument, and a decent burial?" Gentlemen, allow me to say just here that while we may have such persons in the profession who do not respect themselves, either in the United States or in the Dominion of Canada, not enough even to respect the profession, it is not so in England, which we may be proud of; the very country so late in taking hold of the profession, has now set an example, that not only we of the Dominion would do well to follow, but those especially in the United States of America. The English standard of professional status ranks higher than in any part of the world. In the *British Journal of Dental Science* we find it stated that in England the register of dentists is kept by the Medical Council, and the government of the profession practically rests with the medical men. In England, the idea is that reputable dentistry has something to do with medicine. To obtain a place upon the register, the applicant must possess the necessary certificate granted by a competent Examining Board, and it is required that the student or applicant, be he graduate or not, shall sign an agreement, that in case he subsequently attempts to attract business by public advertising or circulars, or shall indulge in any practice considered by the Examining Board unbecoming to the profession, or to a professional man, the diploma shall be cancelled, as was done recently in the case of H. F. Partridge, a graduate of the Royal College of Surgeons in Dublin, Ireland. The Doctor's diploma was cancelled, and his authority to practise dentistry revoked by the Board of Examiners, and sanctioned by the Court of Appeal in England.

Begging pardon for not following more closely an intended outline, I now return to the last part of the seventeenth century and the beginning of the present, in reference to dentistry, and which might be called the bone period. The bone workers and mechanics in those days in England were willing to earn a dollar a day for their services, in constructing plates for those who employed them. These frames or plates, as we may name them, were made from bone, ivory, or hippopotamus' teeth, filed and carved to models made to fit, as well as could be, from impressions of the mouth, and human teeth attached to these frames by means of pins, screws, or otherwise. For a full set, spiral springs were attached to each case to enable them to be worn. Partial cases were either wired or ligated to the natural teeth and allowed to remain oftentimes for years, or until the loss of the natural ones to which they were attached, before these fabrics were removed from the mouth for even comfort and cleanliness. Partial cases were also made from one piece of bone, with the teeth filed, cut and shaped to suit the case. The case, No. 1, will illustrate the latter method, and was made in Halifax, by Dr. L. E. Van Buskirk, in 1822. The case, No. 2, which I now present for your examination, came into my possession twenty-five years ago, and was made for and worn by a lady of wealth and position in this city, long since deceased. This case was made in London about the year 1820 or 1825, and worn until I replaced them with a full set on gold plate with block teeth. Many methods were resorted to to secure and obtain sound and perfect human teeth to attach to the bone plates. Advertisements were inserted in the daily papers in England, offering to purchase perfect teeth from those who were disposed to sell; and many among the poor and unfortunate found sale for those organs, and when a price was agreed upon between the two parties, the teeth were removed.

During the Crimean War, it was said the teeth of some of those slain in battle were removed for the purpose of selling them to dentists for artificial dentures, and when I first commenced to practice in Halifax, in 1860, it was asked by many if natural teeth for artificial sets were used, so general had become the idea that dead people's teeth were the only kind inserted, many objecting and refusing to have sets or partial sets for fear of wearing other people's teeth, as was frequently remarked.

How teeth were removed, or by use of what kind of instruments, we are led to infer that the hawk's bill or turn key, so called, was the instrument then in vogue, as forceps were not in use until the year 1839. The ancient instrument, the turn key, was made with handles at right angles with the shaft, so that both hands could grasp it at once; there were hooks curved and of various sizes, to be attached to the extreme end of the shaft, so as to go over the top of the tooth to be removed, while the bulb of the instrument would be carefully secured and wound with an old handkerchief or a piece of cloth, partly to lessen the pain sure to be produced by using the inside of the jaw and process as a fulcrum when extracting, and particularly to fill the patient's mouth so as to prevent any audible sound during the operation; and if one or even two teeth came forth with a large part of the process, the operator exultingly held it up, exclaiming, "Thank goodness, the jaw is not broken;" while in some cases the operator found, to his sorrow, that the patient had to call in the services of a surgeon to repair damages.

It was my fate in 1843 to have a molar removed with the turn key, my head between an assistant's knees, who stood behind my back, while I was seated on a low stool, and the operator in front performed the terrible deed. One such experience is enough in one lifetime. Instruments for removal of teeth were said to have been found in the ruins of Herculaneum and Pompeii, but as they were made of lead, it was supposed they were more humane in those days than later on, as the lead instruments could be used only for removal of loose or deciduous teeth. I have sometimes wondered what instrument was used for the removal of the Jew's ten teeth in the time of King John, before the money was exacted from him by the king. One of the most primitive and barbarous methods was resorted to in Cornwall in 1849, and the particulars related to me by the Hon. Dr. Wm. Webster, of Kentville. A certain expert, a blacksmith, whose name I forget, near Kentville, in attempting to remove an upper molar for a farmer, used a spike and hammer, and in so doing, fractured so large a portion of the superior maxillary process that the doctor removed two teeth with the process united that had been detached and forced into the palatal portion of the mouth, and only held in position by means of the gum and mucous membrane, and resulting in an opening into the antrum and defective speech for life.

It was in 1839 the AMERICAN JOURNAL OF DENTAL

SCIENCE was first published by Drs. Harris and Parsley, and the American Society of Dental Surgery began its existence in 1840. The first dental school was in Baltimore, and up to 1854 three dental colleges were struggling for an existence, with no instructor in two of them in operative or mechanical dentistry. One of the first graduates of the first class in 1841, was B. Covington Mackall—fifty-nine years ago. Dr. Mackall was living at Elkton, in Maryland, in 1887, enjoying good health, and a member of the Maryland Legislature.

In 1848 the *Dental News-Letter* was first started, now our prized and valuable *Dental Cosmos.*

To America belongs the honor of first establishing colleges, schools and dental journals for the purpose of advancing and educating those who desired to make dentistry a specialty, while it is only during the past six years that England established schools and colleges for dental education, and last, but not least, our own Dominion has not lost sight of the need for educating dentists by also opening dental colleges.

To go back forty years of my own experience in the particular branch as a dentist which I have followed most persistently and closely, and note particularly all the improvements and advancement made since 1852, would not be possible nor even desirable; but let me say here, gentlemen, in spite of all the modern improvements and new appliances, and better facilities for doing good work, I can testify that, even with the so-called crude methods of operating, I can show in my mouth two gold fillings still in excellent condition, made by a Portland dentist, using Abbey's foil and non-serated pluggers, hand pressure, and seated in an ordinary rocking chair, which speaks volumes for honest dentistry, even at that period. Ether and chloroform were not generally used, as ether was introduced in 1847, and Dr. Morton was credited as being the discoverer. Gold, silver and platina plates were used for artificial dentures. Block teeth were manufactured by the leading dentists in Boston, New York and other cities. In Portland, with whom I was studying at that time, as well as Dr. Salmon and Dr. Coffin, manufactured their own blocks, also made continuous gum work mounted on platina plates and gum baked by Dr. Allen's recipe. Single plate and gum teeth were used by others; they were supplied by the old firm of Jones, White & McCurdey. It was not until 1854 that vulcanite rubber was used and introduced. The firat case made in Halifax to my recollection was in 1865, and vulcanized for three hours in a ten gallon vulcanizer. Gutta-percha was also tried, but proved a failure. A case was made on gutta percha plate, single teeth, for a sea captain, ready to sail for the West Indies—result, total collapse after wearing only a few days. In order to use the vulcanite rubber, we were obliged to pay to the patentee an office right of $100, which after some years was abolished.

From 1852 to 1872 advanced strides and improvements continued to be made in dentistry, leading up to more rapid and better success in operative as well as prosthetic dentistry, celluloid plates, the rubber dam, automatic plugger, cohesive gold, dental engines, serated pluggers, hand mallet and numerous other appliances too great in number to be enumerated at the present time. In those days, Boston, Portland, New York, Philadelphia and other large cities of the United States could boast of the highest professional skill and intelligence. Among the leading dentists of those cities were the names of Drs. Tucker, Weatherby, Harvard, Salmon, Parsons, Goodnough, Flagg, Osgood, Alport, Codman, Greenwood, besides a host of others. Quite a number of these gentlemen I had the pleasure of knowing intimately, and was associated with four in business. The reputation acquired by Drs. Harkell and Salmon, with whom I was associated in Boston, and Dr. Parsons, in Portland, stood second to none at that time. The former could not be surpassed in prosthetic dentistry and manufacture of block teeth on gold plates and continuous gum work on platina, as well as partial sets on silver and gold plates. Dr. Salmon is still in practice in Boston, and is the inventor of several dental instruments. Dr. L. P. Harkell's name has become familiar to many down to the present day, and the genial, kind Doctor still follows his profession in Chicago, having given to the profession valuable works on dental prosthesis, and having contributed various articles to our dental journals. A certificate for dental prosthesis is now in my possession, presented by Dr. Harkell while associated with him in 1856, which I prize most highly.

Nitrous oxide gas has been gradually introduced as an anæsthetic agent for the removal of teeth and surgical operations. It was first known as laughing gas. As early as 1849 it was administered at entertainments for the amusement of the public, and its effect on individuals, as given at that time, produced oftentimes strange and peculiar results, showing the disposition very markedly of the individual under its influence, and his pugilistic tendencies. As early as 1850, it fell to my lot, at Wolfville, to test its peculiar qualities previous to an entertainment to be given on the following evening by a certain professor who was travelling the country.

The Colton Dental Association of Boston was the first to use the nitrous oxide gas extensively for the removal of teeth, and later on, Dr. Thomas, of Philadelphia, made a specialty in that way.

From anæsthetics, the average death rates are said to be as follows:

One in 100,000 from Nitrous Oxide Gas.
" 10,000 " Ether.
" 1,000 " Chloroform.

During the last twenty years, from 1872 to 1892, schools, colleges, societies, journals, books and periodicals have largely increased, and the latter more generally read and circulated. All these have a tendency to advance and educate, especially the younger members of the profession, and stimulate the older ones, so as to keep pace with the times.

In looking over the list in the Dental Necrology for the past twenty years, we find some 364 men of more or less prominence in dentistry have passed away, some just entering the profession, others of long standing and residents of the United States, while scores of names not enumerated, in England and the Dominion, have gone to their long home. The Abbeys, Abbots, Allens, Ambler, Atkinson, Parmley, Riggs, Webb, Harvard, Knapp, McQuillan, Kingsley, Neal, Chandler, names familiar to many, all had their day, and each and all have left names to be honored and revered, and whose works still live, and who have left their "footprints on the sands of time."

With all the improvements made in the profession during the past forty years, those who remember what facilities were offered them then in practice, and the present opportunities and methods of working, both in mechanical or prosthetic dentistry, as well as conservative dentistry, have no reason to feel ashamed of their professional skill, and while many who have now passed away have done much to elevate and advance the profession, by their skill and honest worth, as well as by their professional and gentlemanly deportment, gentlemen, let us not forget that to be members of a profession conveys with it certain privileges and rights that should be respected by each and every member. As a Dental Society we should guard jealously the good name of the profession, let us profit by whatever errors or mistakes made in the past, and learn to draw the professional line, as they are doing in England, so that, as professional men, we may become actuated by a true professional spirit—but that day will not be hastened by ridiculing the professional tendencies or aspirations which should be cultivated, or by endeavoring to foster the idea that dentistry is a mere mechanical trade, having no affiliation with medicine, or any right to a professional code of ethics.

May the near future place this Society and every member of the dental profession where its freedom and privileges shall be sustained and guarded as in England, and all advertisements beyond a professional card considered disgraceful and unprofessional in our profession, as in law and medicine, and by those who fill our places forty years hence, may it be said of us, as we can truly say of many in the past, "They labored not in vain and their good works follow them." Let us be united, press on with honest and true principles of right, with hearts within and God overhead.—*Dominion Dental Journal.*

---

ARTICLE II.

